# Assessment of Dog Owner Concern Regarding Peri-operative Nausea and Vomiting and Willingness to Pay for Anti-emetic Treatment

**DOI:** 10.3389/fvets.2019.00264

**Published:** 2019-08-22

**Authors:** Bonnie L. Hay Kraus, Callie Cazlan

**Affiliations:** Department of Veterinary Clinical Sciences, Lloyd Veterinary Medical Center, College of Veterinary Medicine, Iowa State University, Ames, IA, United States

**Keywords:** peri-operative nausea and vomiting, PONV, peri-anesthetic nausea and vomiting, owner anesthesia concerns, opioid associated nausea and vomiting, willingness to pay

## Abstract

**Objective:** The objective of this study was to assess dog owners' concern regarding peri-operative nausea and vomiting, and their willingness to pay for treatment.

**Design:** Descriptive survey.

**Sample:** A survey was administered to 104 dog owning clients with non-emergent surgical (52) or non-surgical (52) appointments at a University teaching hospital.

**Procedure:** Descriptive statistics were calculated. A Mann-Whitney *U* test was used to detect differences between clients expecting their pet to undergo elective general anesthesia and those that did not. A Spearman's Rank Co-efficient was used to correlate predictive data.

**Results:** Ninety-seven (93%) dog owners had at least some worry regarding their dog experiencing nausea associated with opioid analgesics and anesthesia, with 39/104 (37.5%) moderately to very worried. Forty-one owners (39%) would definitely and 59/104 (56.7%) would likely choose treatment to decrease or prevent signs of nausea. Ninety-four owners (90.4%) had at least some worry regarding vomiting, and 48/104 (46%) indicated they were moderately to very worried. Fifty-three owners (51.4%) would definitely and 49/103 (47.6%) would likely choose treatment to prevent vomiting. The median and mean amount owners were willing to pay was 50 and 76.47 USD, respectively. Ninety-five (91.3%) were likely or very likely to opt for treatment if required to arrive 1 h earlier for their appointment. There was no correlation between age, income, or owner's PONV experience with likelihood of choosing treatment but there was a significant positive correlation with the owner's level of education.

**Conclusion:** Canine owners are concerned with their pets experiencing nausea and vomiting in relation to opioid analgesics and anesthesia and are willing to pay and stay the required time for effective treatment.

## Introduction

In dogs, nausea and vomiting associated with anesthesia is most commonly due to the administration of opioid pre-medications. The incidence of vomiting associated with opioids in dogs is 50–75% with morphine (1–6), 44–100% with hydromorphone (1, 7–10), and 33% with oxymorphone (1). The incidence of vomiting can be affected by the specific drug and its lipid solubility profile, the dose and route of administration and concomitant drug administration. Decreasing incidence of vomiting may be observed with higher opioid doses, higher lipid solubility of the opioid drug and prior administration of acepromazine (1, 3, 7, 10–12).

Maropitant^1^, a neurokinin-1 (NK-1) antagonist, is approved by the US Food and Drug Administration to treat and prevent vomiting in dogs and cats. Maropitant prevents binding of the neurotransmitter Substance P (SP) which is found in high concentrations in both the chemoreceptor trigger zone (CTZ) and the vomiting center (VC) and, thus, has been shown to significantly decrease vomiting due to both centrally acting and peripherally acting emetogens and providing broad-spectrum inhibition of vomiting (13). Maropitant has also been shown to be effective in preventing vomiting and signs of nausea when 1.0 mg/kg is administered SC 1 h prior to administration of intramuscular hydromorphone (8).

There are several advantages to avoiding peri-operative vomiting and nausea in veterinary patients. Multiple published studies have implicated perioperative vomiting in dogs as a risk factor for post-operative aspiration pneumonia which is associated with a high mortality rate (14–17). Prevention of concomitant increases in intraocular or intracranial pressure associated with vomiting would also be advantageous to avoid in certain specific patient populations, such as those with glaucoma, eye injuries or intracranial disease. Amelioration of nausea may be responsible for improving anesthetic recovery scores and an earlier return to post-operative feeding, especially in females undergoing ovariohysterectomy (4).

Furthermore, preventing perioperative nausea and vomiting may be considered an animal welfare issue as freedom from discomfort, pain and distress are basic tenants of Brambell's Five Freedoms of animal welfare. Human anesthesia patients report high levels of discomfort, distress and dissatisfaction associated with perioperative nausea and vomiting and consider it to be among the top undesirable anesthetic outcomes (18, 19).

The willingness to pay (WTP) technique has been used in human medicine, when valuing health care interventions and determining patient preferences, especially for therapies that do not directly prolong life but improve the short-term quality of life, such as amelioration of pain and suffering (20). WTP is used to evaluate the maximum amount subjects would be willing to pay to obtain a specific, often intangible benefit including the impact on patient discomfort, anxiety and distress. WTP is based on a simple supply-demand theory of economics where a consumer will pay up to a certain amount for a benefit but, above a maximum WTP, the benefits no longer outweigh the cost. This results in a health outcome valued in monetary terms. WTP studies in humans have found that they would pay between 56 and 100 USD (81.73–145.94 USD in April 2019 USD according to CPI calculator^2^) out of pocket and would allocate the largest amount of money (30 USD out of 100 USD) to avoid vomiting and nausea associated with anesthesia (21, 22). Furthermore, parents would pay ~63 USD (equivalent to 100.12 USD in April 2019^2^) to reduce their child's peri-anesthetic vomiting (23).

The issue of peri-anesthetic nausea and vomiting in veterinary patients has only recently garnered attention. This is likely due to the increasing focus on pain management, including the use of mu-agonist opioids for the treatment of moderate to severe pain and also a focus on patient well-being as a matter of animal welfare. However, to the author's knowledge, there is no information regarding pet owner's attitudes or concerns regarding their pet experiencing these side effects of opioid analgesic medications nor their willingness to pay for treatment to prevent or treat these side effects.

The objective of this study was to survey dog owners to assess their concern regarding their pet experiencing nausea and/or vomiting associated with opioid pain medications and general anesthesia and assess their willingness to pay for treatment. Since ~1 h is required for the prevention of signs of nausea in addition to vomiting, assessment of an owner's willingness to stay or come in early for treatment was also included. A secondary objective was to evaluate demographic data, personal experience with peri-operative nausea and vomiting and their association with the client's WTP.

We hypothesized that ~50% of dog owners would be concerned with their pet experiencing peri-anesthesia nausea and vomiting and would be willing to pay. We also hypothesized that those same owners would spend the required time for anti-nausea/antiemetic treatment for their pet and that the WTP was related to the client's demographic characteristics, specifically education and income.

## Materials and Methods

A survey was administered to dog owning clients of the Iowa State University College of Veterinary Medicine Lloyd Veterinary Medical Center (ISU-LVMC). The survey was formulated based on a similar survey administered to human patients presenting for elective day surgery (Appendix A) (21, 23) Approval was obtained by the Iowa State University Institutional Review Board (IRB ID: 14-254). Informed consent was obtained prior to completion of the survey; clients did not receive any compensation for participation. The participants were required to be at least 18 years of age with a non-emergent appointment for their canine patient at the ISU-LVMC. Clients were not given any written or verbal educational information with regard to the risks or complications of peri-operative vomiting, the specific drug used to prevent vomiting or the real cost. The survey had them consider the following scenario and answer the questions based on this scenario: “*For the purposes of these questions, we would like you to assume that your pet is about to undergo a simple surgical procedure that requires a general anesthetic and use of opioid pain medications*. ***There is a 2–3 in*** ***4 (50–75%) chance that your pet will be sick (nausea and vomiting) after the*** ***pain medication is given*.**” One hundred and twelve (112) clients were asked to participate. One hundred and nine (109) surveys were collected; three clients declined to participate. Five surveys were eliminated from statistical evaluation; four due to lack of signed consent, and one was a duplicate of the same client on two different days (one to drop off the patient for surgery and one to pick up). Fifty-two surveys were completed by clients whose dogs had a non-emergent appointment with the ISU-LVMC small animal services and anticipated elective general anesthesia for a surgical intervention or diagnostic imaging. Fifty-two surveys were completed by clients whose dogs had a non-emergent appointment that did not require general anesthesia. A Mann-Whitney *U* test was used to detect differences in answers to questions 1 through 5 between clients expecting their pet to undergo elective general anesthesia and those that did not. Descriptive statistics were then calculated on the pooled surveys. Spearman's Rank Co-efficient was used to correlate certain demographic data with the likelihood of choosing treatment if recommended by their veterinarian and the cost was 30 USD (Question number 5). Correlation significance was set at *p* = 0.05.

## Results

There was no significant difference between clients expecting their pet to undergo elective anesthesia and clients who did not for questions 1–5 regarding degree of worry about nausea and vomiting and choosing treatment (*p* = 0.146, *p* = 0.176, *p* = 0.196, *p* = 0.679, *p* = 0.945, respectively). Therefore, all surveys were pooled (104) and analyzed together for descriptive statistics and correlation of demographic data.

**Figure 1:**
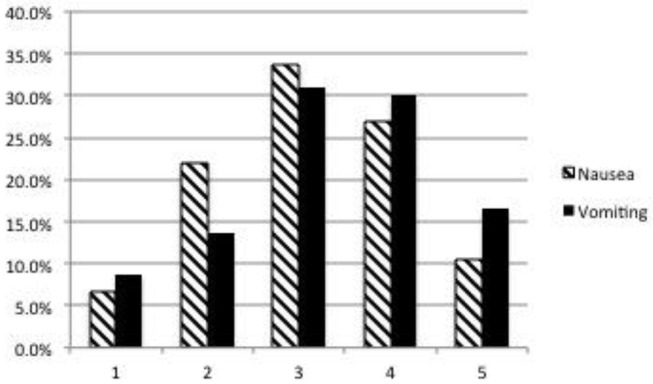
Dog owner's degree of worry about peri-operative nausea and vomiting. 1 = Not at all worried, 5 = Very worried. Respondent answers: Nausea: 1 = 7/104 (6.7%), 2 = 23/104 (22%), 3 = 35/104 (33.7%), 4 = 28/104 (27%), 5 = 11/104 (10.6%). Vomiting: Respondent answers (one client did not answer the question): 1 = 9/103 (8.7%), 2 = 14/103 (13.6%), 3 = 32/103 (31%), 4 = 31/103 (30%), 5 = 17/103 (16.5%).

**Figure 2:**
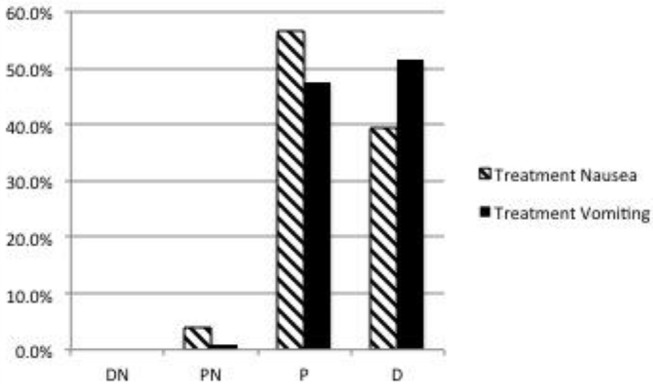
Dog owner's likelihood to choose treatment to prevent nausea and vomiting. D, Definitely; P, Probably; PN, Probably Not; DN, Definitely Not. Respondents answers: Nausea: DN = 0/104 (0%), PN = 4/104 (3.8%), P = 59/104 (56.7%), D = 41/104 (39.4%). Vomiting: Respondent answers (one client did not answer): DN = 0/103 (0%), PN = 1/103 (1.0%), P = 49/103 (47.6%), D = 53/103 (51.4%).

**Figure 3:**
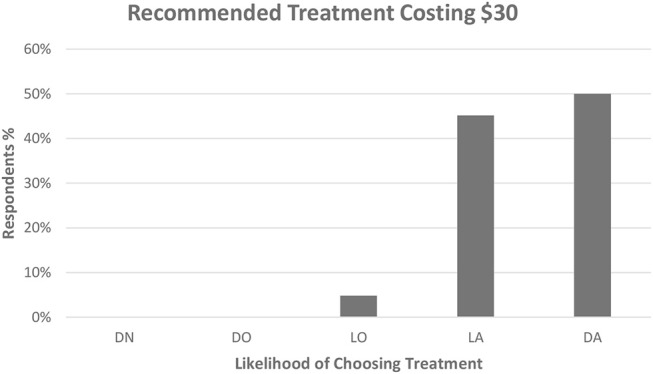
Dog owner's likelihood to choose treatment to prevent vomiting and nausea if their veterinarian recommended treatment and the cost was 30 USD. DN, Definitely not choose treatment; DO, Definitely ask for other options; LO, Likely to ask to other options; LA, Likely to accept treatment; DA, Definitely accept treatment. Respondent answers: DN = 0/104 (0%), DO = 0/104 (0%), LO = 5/104 (4.8%), LA = 47/104 (45.2%), DA = 52/104 (50%).

Ninety-seven (93%) dog owners had at least some worry regarding their dog experiencing nausea in relation to opioid analgesics and anesthesia; only 7/104 (6.7%) were not at all worried. Thirty-nine (37.6%) indicated that they were moderately to very worried (Figure 1). Forty-one owners (39.4%) would definitely and 59/104 (56.7%) would probably choose treatment to lower or prevent signs of nausea (total 96.1%) (Figure 2). Ninety-four owners (91.3%) had at least some worry regarding vomiting and 48/103 (46.6%) indicated they were moderately to very worried; one client did not answer the question (Figure 1). Fifty-three owners (51.4%) would definitely and 49/103 (47.6%) would probably choose treatment to prevent vomiting (total 99%); one client did not answer (Figure 2). If the treatment to reduce nausea and vomiting were recommended by their veterinarian and the cost was 30 USD, 52/104 (50%) would definitely and 47/104 (45.2%) would most likely accept treatment for their pet (total = 95.2%) (Figure 3). When owners were asked the open-ended question of the maximum amount of money they were willing to pay for their pet to receive treatment, the range of responses was from nothing to any amount. Six respondents were willing to pay any amount and 12/104 did not respond (left the answer blank) or indicated “not sure.” Respondents that filled in a specific dollar amount ranged from 20 to 2,000 USD. Of those that answered the question, the most frequent response was 50 USD (38/92). The median and mean amounts that owners were willing to pay was 50 USD and 76.47 USD, respectively.

**Figure 4:**
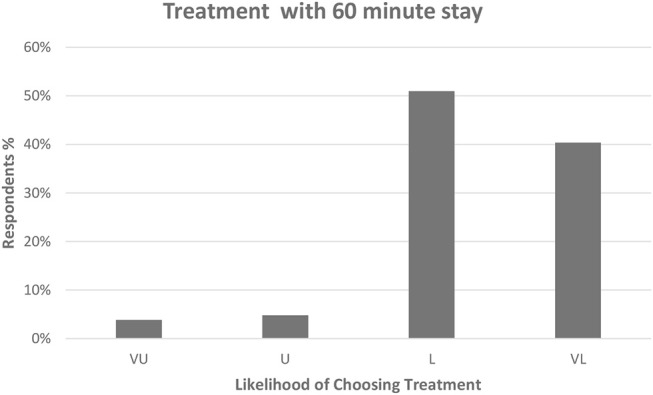
Dog owner's likelihood of choosing treatment if required to arrive 60 min earlier for their appointment. VU, Very Unlikely; U, Unlikely; L, Likely; VL, Very Likely. Respondent answers: VU = 4/104 (3.8%), U = 5/104 (4.8%), L = 53/104 (51%), VL = 42/104 (40.4%).

If owners were required to bring their pet into the veterinary clinic 60 minutes earlier in order to receive the treatment, 53/104 (51%) were likely and 42/104 (40.4%) were very likely (total of 95/104, 91.4%) to still choose this treatment option. Only 9/104 (8.7%) were unlikely or very unlikely to choose treatment due to this additional time commitment (Figure 4).

When owners were asked the open-ended question of the maximum amount of time they were willing to spend to receive treatment, the range of responses was from nothing to any amount of time. Thirteen clients did not answer the question and fourteen indicated that they would spend any amount of time necessary for their pet to receive the treatment. One client wrote several hours, and this was interpreted as >2 h and, therefore, 3 h was used for statistical evaluation. Two clients wrote “1 day” and this was interpreted as 24 h or 1,440 min for statistical analysis. The mean, median and mode for the maximum time clients would spend for their pet to receive treatment to prevent peri-operative nausea and vomiting was 144, 75, and 60 min, respectively.

Demographic and personal data with regard to gender, marital status, children, other pets, age, education level, household income, work situation, personal experience with nausea and vomiting associated with surgery or anesthesia in themselves, a child or another pet are presented in Table 1. The majority of respondents were married (70%) women (63.5%). Respondents were well-educated with the majority (88%) having at least some college education. Fifty-seven percent were college graduates or had post-graduate work or degrees. The median income was 75–100,000 USD; the mode for income was 100–200,000 USD. Over 50% of respondents had personal experience with peri-anesthetic nausea and vomiting. There was no significant correlation between age, income, nor experience with owner's peri-operative nausea and vomiting (either themselves, their child or another pet) with the likelihood of choosing treatment if it were recommended by their veterinarian and the cost was 30 USD. However, there was a significant positive correlation with the owner's level of education and the likelihood of choosing treatment (Table 2).

**Table 1:** Demographic and personal information of clients completing the survey.

**Variable**	**Total number of respondents**	**%**
**Gender**		
Male	38/104	36.5
Female	66/104	63.5
**Married**		
Yes	73/104	70.2
No	31/104	29.8
**Children**		
Yes	57/104	54.8
No	47/104	45.2
**Additional pets**		
Yes^*^	66/104	63.5
No	38/104	36.5
*Clients that indicated yes^**#**^		
Additional dogs (1–13)	50/66	75.8
Cats (1–13)	35/66	53
**Age (5 clients did not answer)**		
18–30	23/99	23
31–40	18/99	18
41–50	18/99	18
51–65	34/99	34
>65	6/99	6
**Level of education (6 clients did not answer)**		
Grade school	0	0
Some high school	3/98	3
High school graduate	9/98	9.2
Some college/Jr. college degree	30/98	30.6
College graduate	29/98	29.6
Post-graduate work/degree	27/98	27.6
Income (11 clients did not answer)^**^		
<15,000 USD	5/93	5.4
15–30,000 USD	10/93	10.8
30–45,000 USD	9/93	9.7
45–60,000 USD	15/93	16.1
60–75,000 USD	7/93	7.5
75–100,000 USD	19/93	20.4
100–200,000 USD	23/93	24.7
>200,000 USD	5/93	5.4
**Work situation (6 clients did not answer)**		
Full time (>30 h/week)	55/98	56
Part time (<30 h/week)	11/98	11.2
Student	9/98	9.2%
Student/Part time worker	3/98	3.1
Working at home	6/98	6.1
Not working	7/98	7.1
Retired	7/98	7.1
**Personal experience with PONV**		
**Self (5 clients did not answer)**		
Yes	50/99	50.5
No	49/99	49.5
**Child (10 clients did not answer)**		
Yes	13/41	31.7
No	28/41	68.4
Not applicable	53/94	56.4
**Other Pets (14 clients did not answer)**		
Yes	11/55	20
No	44/55	80
Not applicable	35/90	38.9

# *Thirteen clients indicated that their additional pets were categorized as “Other pets” (number of respondents): rabbits (4), guinea pig (1), goats (1), horses (1), birds (6), turtle (2), fish (2), chickens (2), lizard (2)*.

* Clients that indicated “yes” to question 18 in Appendix A.

** *Median income: 75–100,000 USD and the mode for income was 100–200,000 USD*.

**Table 2:** Spearman correlation coefficients between willingness to pay and other variables obtained in the questionnaire.

**Variable**	**Correlation**	***p*-Value**
Age (*n* = 99)	0.00919	0.92824
Income (*n* = 93)	−0.1508	0.15134
Education level (*n* = 98)	0.22162	0.0283^*^
**Experience with PONV**		
Self (*n* = 99)	−0.0285	0.77946
Child (*n* = 41)	−0.01826	0.91096
Other pets (*n* = 90)	−0.11788	0.38688

* *Correlation is significant at p = 0.05 (two-tailed)*.

## Discussion

This survey of dog owners indicates that the overwhelming majority of dog owners in this survey are concerned with their pet experiencing nausea and vomiting associated with anesthesia and would be willing to pay for treatment, especially if recommended by their veterinarian. The incidence of vomiting associated with anesthesia in human patients is ~30% and the incidence of nausea is about 50%, however, both can be as high as 80% in a sub-set of high-risk patients (19). The causes are thought to be volatile anesthetics, nitrous oxide and opioids (19). Similar incidences for vomiting have been reported for dogs receiving commonly used opioid analgesics (1–10). Human anesthesiologists rank pain, nausea and vomiting as the top low morbidity clinical anesthesia outcomes they believe to be important to avoid (24). Likewise, surveys of human patients consistently rank pain, vomiting and nausea as the top three most undesirable post-operative outcomes (22, 25–27). The desire to avoid nausea and vomiting associated with anesthesia is a universal inclination amongst human beings. Regardless of differences in culture, socioeconomics or healthcare structure, patients in the USA, Europe, Singapore and Turkey all endeavor to avoid nausea and vomiting as a side effect of anesthesia (21, 27, 28). Considering the degree to which humans desire to avoid experiencing nausea and vomiting associated with anesthesia, the authors anticipated that dog owners would have at least some concern regarding their pet experiencing these side effects. However, the prevalence and degree to which dog owners were concerned regarding their pet experiencing peri-anesthetic nausea (93%) and vomiting (91%) far exceeded the author's expectations. This level of concern is comparable to, but even higher, than the degree of worry expressed by parents regarding their child experiencing peri-operative nausea and vomiting where ~70% of parents expressed at least some degree of worry and 24% were very worried (23). Parents ranked vomiting as the most undesirable side effect (pain ranked as second) for their children undergoing surgical procedures and were significantly less satisfied with their child's perioperative care if their child did experience nausea and vomiting (29, 30). According to the recent American Pet Products Association (APPA) pet owner survey, 59% of dog owners consider their pet like a child or family member (31). It would stand to reason that dog owners' humanization of their pets and concern for their welfare are drivers for the amount of concern they have for their pet experiencing sensations and side effects that they themselves would distinctly like to avoid. This sentiment is also reflected in that 96 and 99% of owners would probably or definitely choose treatment for the prevention of nausea and vomiting in their canine pets.

Over 95% of owners would most likely or definitely choose treatment to reduce the likelihood of nausea and vomiting if it were recommended by their veterinarian and would cost 30 USD. According to the APPA survey, the majority of dog owners (60%) stated that their veterinarian is their major source of pet related information (31). The AVMA Pet ownership and demographics source book (PDS) also reinforces the importance of the owner/veterinarian relationship. The majority (85–90%) of owners state that they have a “regular” veterinarian whom they prefer and cite knowledge and quality of care, in addition to the kind, compassionate handling of their pets as the top reasons for their preference (32). The results of the current study should assure veterinarians that awareness of the risks and negative effects of peri-anesthetic nausea and vomiting and the benefits of its prevention will be viewed favorably by their clients. Additionally, dog owners will most likely choose treatment for the prevention of nausea and vomiting if given a choice, or be willing to pay for prevention if it is incorporated into pre-anesthetic medication protocols.

WTP studies have been used in the human adult and pediatric surgical populations to assess the patients' and the parents' of patients perspectives regarding avoidance of anesthesia side effects or outcomes, such as nausea and vomiting. Over 95% of dog owners would most likely or definitely choose treatment to reduce the likelihood of nausea and vomiting if it were recommended by their veterinarian and would cost 30 USD. The median and mean maximum amount that dog owners were willing to pay for their pet to receive treatment to prevent nausea and vomiting was 50 and 76.47 USD which is equivalent to 54.62 and 83.54 USD (as of April 2019^2^). These dollar amounts are slightly lower than they would spend to avoid nausea and vomiting in themselves but comparable to what they would spend to avoid nausea and vomiting in their children. Human patients are WTP 56 USD (81.73 USD as of April 2019^2^) out of pocket for an effective anti-emetic, whereas patients who actually experienced nausea and vomiting were WTP more; 73 and 100 USD, respectively (106.54 and 145.94 USD as of April 2019^2^) (21). Women who have undergone gynecological laparoscopy were WTP 117 ± 82 USD (185.02 ± 129.62 USD as of April 2019^2^) for prophylactic anti-emetic treatment to prevent nausea and vomiting if they were to undergo a similar procedure in the future (33). Pre-operative human patients ranked vomiting as the as the most undesirable anesthesia outcome and allotted the largest amount (30 USD) of a theoretical 100 USD to avoid these outcomes (22). The WTP for the effective prevention of nausea and vomiting associated with anesthesia is similar across human cultures, socioeconomics and healthcare structures; German patients were willing to pay the equivalent of ~86.52 USD and Turkish patients 90.51 USD (as of May 2019^3^,^4^) (28). Patients who had experienced nausea and/or vomiting post-operatively were willing to pay larger amounts; 127.79 USD in Germany and 131.78 USD in Turkey, respectively (as of May 2019^3^,^4^) (28). Like patients in the US and Europe, patients from Singapore ranked vomiting and nausea, along with pain as the top 3 adverse outcomes and allotted an equal portion of a hypothetical 100 USD to avoid vomiting and nausea as they would to avoid pain (50 USD each) (27).

Parents rank vomiting as the most unwanted side effect, followed by pain and then nausea (29). They also allocated the most amount of money for its prevention (41.93 USD out of 100 USD), whereas parents allotted 33.48 USD out of 100 USD to the prevention of pain (29). Another study found that parents are WTP a median value of ~63.31 USD (100.12 USD as of April 2019^2^) for a reduction in post-operative emesis in their children (23).

A possible limitation of this survey is that question number 5 could have resulted in a starting point bias for question number 6 which asked for the maximum amount that owners were willing to pay (Appendix A). There are three ways to obtain WTP values. Bidding questions, closed-end questions and open-ended questions (23). In bidding questions, various values are suggested to the respondents who then choose the maximum value they are WTP (23). With this method, the responses are directly dependent on the suggested values. Closed ended questions will suggest one value to respondents who can either accept or decline it (23). Open ended questions ask respondents for the maximum they are WTP without any suggested values. It is known that respondents find these types of questions difficult to answer (23). Our survey used a combination of a closed ended question (number 5) and an open-ended question (number 6) (Appendix A). The closed ended question suggested 30 USD as the cost of the treatment and included a recommendation from the veterinarian. At the time of this survey, the list price of injectable Cerenia^®^ was 119.60 USD (~6 USD/ml)^5^, therefore 30 USD was 2.5 times the cost of treatment of a 20 kg dog. Over 95% of dog owners would likely or definitely be WTP this amount for their pet to avoid nausea and vomiting if their veterinarian recommended it. The open-ended question yielded a very wide range of responses (0 USD to any amount) and dollar amounts from 20 to 2,000 USD. Approximately 12% of respondents left this question blank or wrote “not sure.” Some respondents that wrote 0 USD, indicated that they would definitely choose treatment if it cost 30 USD in the previous question. The mean, median and mode of the maximum amount that owners stated they would pay was 76.47, 50, and 50 USD, respectively (83.54 and 54.62 USD in today's dollars as of April 2019^2^). These responses seem to confirm the difficulty that respondents have in answering open ended WTP questions. Despite this difficulty and possible starting point bias of questions 5 and 6, it seems reasonable to conclude that the amount these owners were willing to pay is substantially higher than the actual cost of the drug. It is standard practice at this institution to include an anti-emetic in all canine anesthetic patients; therefore, it is unknown if these owners would have declined the treatment if asked or if results would differ in different practice environments.

In addition to WTP for treatment, another perceived barrier to owners choosing treatment for prevention of vomiting and nausea might be the time needed for drug effectiveness. Studies indicate that, although vomiting is prevented when the opioid hydromorphone is administered 30 min after SC maropitant, a full 60 min is required to significantly decrease signs of nausea (8, 34). Our survey indicated that the overwhelming majority of owners (91.4%) indicated that they would be likely or very likely to choose treatment despite being required to arrive 60 min earlier for their appointment. Responses to the open-ended question regarding the maximum amount owners were willing to spend for their pet to receive treatment confirm this with owners willing to spend between one and 2.4 h for treatment. Questions 7 and 8 (Appendix A) relating to owners' willingness to stay for treatment may have been prone to the same starting point bias and difficulties with open ended questions as the WTP questions. However, it seems clear that over 90% of dog owners would be willing to stay at least 60 min for effective prevention of nausea and vomiting in their canine pets.

WTP studies often include demographic data for correlation analysis, as was done in our survey. The majority of respondents were women (~64%). Most were married (70%) with children (~55%). According to the U.S. Department of Labor, most family caregivers are women and mothers, in particular, make 80 percent of health care decisions in the United States (35). Since women have a leading role in family health care, it may be fair to assume that this influence may extend to the veterinary care of family pets. The female gender is also the strongest patient-specific predictor for the occurrence of nausea and vomiting associated with anesthesia in human patients (19) and has been shown to have a positive correlation between the respondent and WTP (26). Women in Singapore rank avoidance of nausea as more important compared to men, however, this did not translate into a difference in terms of the patient's WTP (27).

Sixty-four percent of respondents in our survey stated they had additional pets which is higher than reported in the AAPA survey (46%) (31). However, the AAPA survey did not account for owners who owned more than one of the same type of pet. Seventy-six percent of owners in our survey owned additional dogs and 53% owned a cat in addition to their dog. According to the AAPA survey, 46% of current pet-owning household have multiple types of pets and the combination of dogs and cats is the most common (32% of pet owners) (31).

The age range of respondents was fairly evenly distributed amongst the offered age groups, although the highest age ranges were the 18–30 and the 51–65 age groups (Table 1). A high percentage of 18–30 years-olds likely reflects both city and dog ownership demographics. The city of Ames Iowa is a college town with a median age of 23.5^6^ years, which is significantly younger than the median of the entire US, which is 38 years (36). Additionally, the most recent AAPA survey revealed a shift in generational makeup of dog ownership. Thirty-eight percent (38%) of dog owners are now of the Gen Y generation (24–38 years of age) and have supplanted “baby boomers” as the largest group of dog owners (31). In our survey, the highest age group of respondents was the 51–65 years old group and likely reflects the demographics of dog ownership where 31% of dog owners are of the “baby boomer” generation (ages 54–74) (31). Several human WTP studies have documented a positive correlation between patient age and WTP, however, we found no significant correlation with age of the respondent and the likelihood of choosing treatment (21, 37).

It is clear from the demographic data that the clientele of the Iowa State College of Veterinary Medicine Lloyd Veterinary Medical Center are highly educated, with close to 88% having at least some college education and over 57% having earned college degrees and/or graduate studies or degrees. This is reflective of the population of the city of Ames Iowa where over 97% have at least a high school education, 63.4% have a bachelor's degree or higher and 30.8% hold graduate or professional degrees^6^. The percentage of respondents with bachelor's degrees is similar to the US average (32.5%) but the respondents with post-graduate work or advanced degrees (27.6%) is more than twice the US average of 12% (38).

We found a significant positive correlation with the owner's level of education and the likelihood of choosing treatment. Kerger et al. also found a positive correlation between WTP and education level; patients with some college or college degree were eight times as likely to pay more than the equivalent of 86.52 USD (as of May 2019^3,4^) for an effective anti-emetic (28). Look et al. found that avoidance of nausea was ranked as more important by respondents with more than 6 years of education compared to patients with <6 years of education (27). However, this was not accompanied by a difference in their WTP (26). There was also no correlation between parent's WTP for an effective antiemetic for their child and the parent's level of education (23).

The median income of respondents in this survey was 75–100,000 USD which is well above the median income for household income for the city of Ames Iowa (43,737 USD)^7^, the state of Iowa (56,570 USD) (39), and the US (57,652 USD) (39). Statistics from the US Department of Labor delineate a clear relationship between level of education and earnings. People with the highest levels of educational attainment, such as doctoral and professional degrees earn more than three times those with the lowest educational level (40). Therefore, the higher income level may be reflective of the high degree of education of the respondents in this survey. It is unclear if the higher income is indicative of dog or pet owners in general or of clientele visiting specialty clinics, such as ISU-LVMC, in particular. There was no significant correlation between respondent income and their WTP for treatment to prevent nausea and vomiting in their canine pet. This is in agreement with Kerger et al., in which household income did not play a significant role in determining a patient's WTP. However, several other studies have documented a positive correlation between income and WTP (21, 23, 37). In fact, equity considerations are one of the primary criticisms of WTP studies; patients with lower income may be more tolerant of low morbidity outcomes.

Approximately 50% of respondents had personal experience with nausea and vomiting associated with anesthesia or surgery and 20% and ~32% had another pet or child who experienced nausea and vomiting associated with anesthesia or surgery. There was no correlation between the owner's experience with nausea and vomiting (either themselves, their child or another pet) with their likelihood of choosing treatment. This is in contrast to several WTP studies in people where a positive correlation was found between a patient's past or present experience with nausea and vomiting associated with anesthesia and surgery and their WTP (21, 28). However, Macario did not find a positive relationship between the patient's previous experience with nausea and vomiting and their ranking of it as an undesirable outcome (22). Diez also found no correlation with parent's WTP and the child's previous experience with nausea and vomiting (23). This may reflect a notion that the fear or anticipation of nausea and vomiting is equal to the actual experience.

A limitation of this survey is that participants were confined to one mid-western US university teaching hospital. Dog owner attitudes and WTP for treatment may differ in other geographical areas and/or with different demographical characteristics and further multi-center studies are needed. However, WTP for prevention of nausea and vomiting in human anesthesia patients has been found to be similar across countries, cultures and socioeconomic and healthcare structures; therefore, perhaps the population in this study may serve as a microcosm of the greater dog owner population until further studies are completed.

There is a vast availability of health-care information available to today's veterinary health-care consumers leading to better education and perhaps more interest in the details of their pet's care and outcome. The survey demonstrates that the overwhelming majority of dog owners are concerned with their pets experiencing nausea and vomiting in relation to opioid analgesics and anesthesia and are willing to pay and stay the required time for effective treatment. Understanding owner concerns may facilitate the individualization of the anesthetic management of veterinary patients. Veterinary practitioners who target management of these adverse side effects may improve owner satisfaction with the anesthetic care of their canine companions.

## Data Availability

The datasets generated for this study are available on request to the corresponding author.

## Ethics Statement

Approval was obtained by the Iowa State University Institutional Review Board (IRB ID: 14-254). Informed consent was obtained prior to completion of the survey; clients did not receive any compensation for participation. The participants were required to be at least 18 years of age with a non-emergent appointment for their canine patient at the ISU-LVMC.

## Author's Note

Abstract presented at the American College of Veterinary Anesthesia and Analgesia Annual Meeting (in conjunction with the International Veterinary Emergency and Critical Care Symposium), Gaylord National Convention Center, 201 Waterfront St., National Harbor, MD 20745, September 18, 2015.

## Author Contributions

BK: responsible for the study concept and design, formulation of survey, analysis of data, and writing of manuscript. CC: administration of survey and collection of data, assistance with analysis of data, and editing of manuscript.

### Conflict of Interest Statement

BK received research support from Zoetis, primarily in the form of product donations for previous studies involving Cerenia®. BK served on an advisory board for Zoetis. However, the author confirms that the idea, design and all aspects of this survey study originated with the author. The remaining author declares that the research was conducted in the absence of any commercial or financial relationships that could be construed as a potential conflict of interest.

## Footnotes

^1^Cerenia^®^ (maropitant citrate), Zoetis, Parsippany, NJ.

^2^Bureau of Labor Statistics Consumer Price Index inflation calculator: https://www.bls.gov/data/inflation_calculator.htm.

^3^Inflation Calculator, euro: https://www.inflationtool.com/euro.

^4^Exchange calculator euro to USD: https://www.currency-calc.com/.

^5^Zoetis 2019 Companion Animal Products Veterinary Price List. The current list price of injectable Cerenia® is $155.90 (~$7.80/ml).

^6^City data for Ames, IA: http://www.city-data.com/city/Ames-Iowa.html.

^7^City data for Ames, IA: http://www.city-data.com/income/income-Ames-Iowa.html.
