# Supplementary material for: Assessment of Dog Owner Concern Regarding Peri-operative Nausea and Vomiting and Willingness to Pay for Anti-emetic Treatment
Source: Front Vet Sci. 2019 Aug 22;6:264. doi: 10.3389/fvets.2019.00264 (PMC6714863; doi:10.3389/fvets.2019.00264)
Supplement: Supplementary file 1 [file Data_Sheet_1.pdf]

Appendix A

Canine Client Survey

Inclusion Criteria:
Dog owners (> 18 years of age) with a non-emergency appointment at the Lloyd Veterinary Medical Center

For the purposes of these questions, we would like you to assume that your pet is about to undergo a simple surgical procedure that requires a general anesthetic and use of opioid pain medications. ***There is a 2-3 in 4 (50-75%) chance that your pet will be sick (nausea and vomiting) after the pain medication is given.***

1. On a scale of 1 to 5 where 1 is not at all worried and 5 is very worried, how worried are you that your pet will experience nausea due to the pain medication? (Circle one)

1 2 3 4 5

2. If a new treatment were available that would reduce your pet's likelihood of experiencing nausea to less than ten percent (<10%), would you choose this treatment option?

Definitely Not______ Probably Not________ Probably______ Definitely______

3. On a scale of 1 to 5 where 1 is not at all worried and 5 is very worried, how worried are you that your pet will experience vomiting due to the pain medication? (Circle one)

1 2 3 4 5

4. If a new treatment were available that would reduce your pet's likelihood of vomiting to zero (0%), would you choose this treatment option?

Definitely Not______ Probably Not________ Probably______ Definitely______

5. If this new treatment, which reduces the likelihood of experiencing nausea and reduces likelihood of vomiting due to pain medication, was recommended by your veterinarian and would cost $30, which of the following statements best describes your response in this situation?
- ☐ I would definitely accept this treatment for my pet
- ☐ I would likely accept this treatment for my pet
- ☐ I would likely ask my veterinarian about other options
- ☐ I would definitely ask my veterinarian for another option
- ☐ I would definitely NOT accept this treatment for my pet

6. What is the maximum amount of money you would be prepared to pay for your pet to receive this new treatment? _________________

7. If you had to drop off your pet 60 minutes earlier at your veterinarian to receive this treatment, how likely would *you be to choose this new treatment option?*

Very Unlikely______ Unlikely________ Likely______ Very Likely______

8. What is the maximum amount of time you would be prepared to drop your pet off before a surgery in order to receive this treatment? ____________

9. Has your pet ever had surgery and/or anesthesia before? Yes______ No_______

10. Did your pet experience nausea in relation to his/her previous anesthesia/surgery?

Yes______ No______ Don't know ________

11. Did your pet experience vomiting in relation to the pain medication received after surgery?
Yes______ No______ Don't know_________

12. What is the nature of your dog's visit to the Lloyd Veterinary Medical Center today?

Routine exam/vaccinations	_________	Neurologic problem	___________
Medical problem	_________	Eye problem	___________
Surgical problem	_________	Skin problem	___________
Other	_________		

13. Is your pet being admitted today to the Lloyd Veterinary Medical Center for surgery or a diagnostic procedure that requires anesthesia?
Yes ___________ No____________

14. If you answered Yes to #13, would you choose to have your pet receive this treatment prior to anesthesia?
Yes ___________ No ____________

Now we would like to ask you a few questions about yourself. The answers that you give are absolutely confidential and will not be discussed in association with your name. In order for us to understand your answers, we need to know a little about your background.

15. Male _______ Female________

16. Are you married? Yes_______ No_______

17. Do you have any children? Yes ________ No ________

18. Do you have other pets? Yes _______ No _______
If yes, please explain:
other dogs Yes _____ How many? ________
cats Yes _____ How many? _______
other pets, please list:____

19. What is your age?

18–24 _____ 25–30______ 31–40______ 41–50_____ 51–65______ Over 65______

20. What was the last grade of school that you completed?

Grade school or less (grades 0–8) __________
Some high school (9–12) _________
High school graduate (12) _________
Some college or junior college (2 yr degree) _________
College graduate __________
Post graduate work or degree __________

21. What is your approximate household income?

Under $15,000	________	$60,000 - $75,000	_________
$15,000–$30,000	________	$75,000 - $100,000	_________
$30,000–$45,000	________	$100,000 - $200,000	_________
$45,000–$60,000	________	Over $200,000	_________

22. Which category best describes your situation?

Full time worker (30 or more hours a week) _______
Part time worker (under 30 hours a week) ________
Working at home ________
Student ________
Not working for other reason ________

23. Have you yourself ever experienced nausea and vomiting associated with surgery or anesthesia?

Yes_____ NO_____

24. Have your children or other pets ever experienced nausea and vomiting associated with surgery or anesthesia?

Children Yes ______ No _______ Not applicable _______
Other pets Yes ______ No _______ Not applicable _______

THAT'S ALL! We really appreciate that you've taken the time to help us with our survey.
